# Book Reviews

**Published:** 1895-03

**Authors:** 


### Book Reviews.

Medical Jurisprudence, Forensic Medicine and Toxicology. By R. A. Witthaus, A. M., M. D., Professor of Chemistry, Physics and Hygiene, in the University of the City of New York, etc., etc., and Tracy C. Becker, A. B., LL. B., Counselor-at-Law and Professor of Criminal Law and Medical Jurisprudence in the University of Buffalo. In four volumes. Volume II. Large 8vo, 751 pages, illustrated with wood-cuts and three plates, two of them in colors. Price, in muslin, $5.00; in brown sheep and in law style, $6.00 per volume. Sold by subscription only. New York: William Wood & Co. 1894.

The second volume of this important work justifies the opinion previously expressed in regard to it in these columns. The second division, forensic medicine, is continued and the sub-head, bio-thanatological, taken up. The first section, consisting of eighty-two pages, is devoted to the examination of blood and other stains, and is written by Edward S. Wood, professor of chemistry in Harvard University medical school. This subject, next to that of poisons, constitutes one of the most important presented to the chemical expert in medico-legal cases. If the case should prove to be one of homicide, the greatest interest especially surrounds blood stains. The several methods employed for the identification of blood stains are here considered and are, first, chemical; second, optical; and third, microscopical. All these methods are here treated with much elaboration and are illustrated by numerous photographs, showing the difference between human blood corpuscles and those of various animals.

Closely allied to the foregoing is the examination of hair, to which a brief section is devoted and which is treated of by the same author.

The third section of the book, consisting of 125 pages, considers abortion and infanticide. These subjects are elaborately dealt with by J. Chalmers Cameron, consulting physician to the Montreal general hospital. There is probably more concealed crime of which offenders go unpunished in these two departments of criminal jurisprudence than in any others. The difficulties of differentiation between criminal and natural abortion, it must be confessed, are many, and the incentives to concealment are strong.

The physician is often called upon to decide, as an expert, by an examination of the body, whether death is from infanticide. He is confronted by four problems, an intelligent solution of which will clear up many a doubtful case, and often prevent an unjust accusation. These are: first, was the child born alive? Second, if so, how long did it live after birth? Third, was death due to natural causes or to violence? Fourth, was the violence accidental or criminal? A careful reading of this section will prove of great service to the medical witness.

A short chapter is devoted to the determination of survivorship, which is written jointly by Tracy C. Becker, one of the editors, and John Parmenter, professor of anatomy and adjunct professor of clinical surgery in Buffalo University medical college. Mr. Becker treats of the legal side of the subject, while Dr. Parmenter discourses upon its medical aspect. This brief section is full of interesting thought.

We have now reached the second grand division of the work, forensic medicine. The first chapter considers a biological question—namely, when medical examination or other inspection of living human body is required or permitted by courts of law, that is also written by Mr. Becker, who discusses the subject from the criminal and civil points of view.

The next section of the work considers pregnancy, labor and the puerperal state, to which 116 pages are devoted, and it is written by J. Clifton Edgar, associate professor of obstetrics in the University of the City of New York. The interesting subjects relating to feigned pregnancy, unconscious impregnation, and delivery are here discussed with intelligence and clearness. Can copulation and impregnation occur in a woman, without her knowledge, during hypnotic sleep? is a question for the believers in hypnotism to discuss. We are willing to admit that a woman may be unconscious of her pregnant state, but we are not ready to admit that copulation can take place without her knowledge.

The next subject discussed is that of sexual incapacity in its medico-legal relations, and is written by Irving C. Rosse, professor of nervous diseases in Georgetown University. The various questions relating to impotence and sterility are herein set forth with vigorous and forceful interest.

From time immemorial the medico-legal consideration of rape has possessed great interest to the medical jurist, but never, in our view, has it been discussed more intelligently than in this volume by J. Clifton Edgar, whose title we have before given, and James C. Johnson, assistant in the department of skin and venereal diseases at the New York Hospital. This chapter is excellently illustrated by chromo-lithographs and engravings, and is full of illustrative cases demonstrating the several problems pertaining to the subject.

The next chapter is devoted to the consideration of unnatural crimes, and is also written by Irving C. Rosse. Here are detailed the most disgusting practices relating to sexual inversion, bestiality and other like abhorrent crimes.

Let us quickly turn from the foregoing to the next section, which deals with railway injuries, their clinical and medico-legal features, which is written by W. B. Outten, dean and professor of the principles and practice of surgery in the Beaumont Hospital Medical College, St. Louis. In the 176 pages allotted to this important medico-legal question, Dr. Outten has given us more definite information than before has ever been printed in a single monograph on this subject. These cases are oftener contested in the courts than any others, and every physician needs to inform himself thereon with the greatest care and intelligent thought.

This admirable volume closes with a brief chapter on simulated diseases, written by W. Thornton Parker, formerly medical examiner of Rhode Island. The book, like the preceding volume, is full of references and contains a table of cases cited by legal authors in volumes I. and II. It is well indexed and fully sustains the claims made for the work in its prospectus, as well as the fulsome praise that it has received at the hands of reviewers.

---

A CLINICAL MANUAL OF DISEASES OF THE EYE, including a Sketch of its Anatomy. By D. B. ST. JOHN ROOSA, M. D., LL. D., Professor of Diseases of the Eye and Ear in the New York Post-Graduate Medical School and Hospital; formerly Professor of Diseases of the Eye in the University of the City of New York, and in the University of Vermont; Consulting Surgeon to the Brooklyn Eye and Ear Hospital; President of the New York Academy of Medicine; Honorary Member of the Medico-Chirurgical Society of Edinburgh; Honorary Fellow of the Academy of Medicine of Havana, Cuba, etc., etc. Illustrated by 178 engravings and two chromo-lithographic plates. Octavo, pp. xx.—622. New York: Wm. Wood & Co. 1894. Price, cloth, $5.00; leather, $6.00.

This manual is the production of a facile writer, an experienced practitioner and an independent mind. It is the outcome of a motive which, in the author's own words, is as follows:

> This book has not been written because the author supposed, for an instant, that there were not already in the English tongue many excellent treatises on diseases of the eye. It is presented to the profession because I have not deemed the debt I owe to it could be even approximately satisfied, nor my own reputation as a teacher, whatever that may be, justly settled unless I presented in a permanent and accessible form some of the results, with their personal coloring of my long experience both in hospital and private practice, in ophthalmic disease and therapeutics.

In other words, the author had a message to deliver to the medical profession and he desired to secure such delivery beyond all peradventure of loss. The "manual" was selected as the best means of doing this and at the same time as the best channel by which to reach the largest number.

We have here a very complete description of diseases of the eye and their treatment, written in the author's well-known easy and interesting style, into which is incorporated such personal views and practices as he desires shall be preserved and perpetuated. These latter are based upon a broad and well-digested experience and must serve at least to partially neutralize what are considered by the more conservative ophthalmologists as extreme and unsustained claims of certain practitioners, particularly in regard to the frequency, effects and treatment of inequilibrium of the ocular muscles.

The work is divided into four parts, as follows :

Part I.—Sketch of the anatomy and physiology of the various parts of the eye and its appendages.

Part II.—The relative frequency of different diseases of the eye, methods of examination, therapeutics and surgery of the eye.

Part III.—Diseases of the eyelids, the lachrymal apparatus, the conjunctiva, eyeball and orbit.

Part IV.—Conditions of the eye requiring glasses—errors of refraction and accommodation, strabismus, affections of the ocular muscles.

In part first the author gives a good description of the anatomy and physiology of the eye and its various parts.

Part second explains the methods of examination, including the use of the ophthalmoscope and the author's favorite instrument, the ophthalmometer, the principles of treatment and remedies applicable, and concludes with a chapter on surgical operations on the eye. A chapter is also devoted to the relative frequency of the various affections of the eye. In this part the shadow-test is referred to, but is passed over with a brief historical sketch and a very short description of its use. Evidently the author has but little use for it in his own practice. If the description of operations of the eye are to be separated from that of the conditions to which they apply and belong, it is better, in our opinion, to place it before rather than after the latter, as Dr. Roosa has done.

Part third takes up the diseases of the eye in the following order : Eyelids, lachrymal apparatus, conjunctiva, cornea, sclera, iris, ciliary body, choroid, retina, vitreous humor, glaucoma, crystalline lens and orbit. This part gives an excellent survey of diseases of the eye and represents, in the main, the latest and the best in regard to ocular therapeutics.

Part fourth considers errors of refraction and affections of the ocular muscles. It is here that the author asserts his individuality and expresses his convictions in no unmistakable terms. He ignores, almost entirely, muscular " insufficiencies," their nomenclature (Stevens) and effects and simply regards them as symptoms or effects whose cause lies for the most part in errors of refraction. He says :

> Of late years, too much stress has been laid upon the conditions of the eyes in producing constitutional symptoms. Certain constitutional conditions that involve accommodative fatigue, or fatigue of the ciliary muscles, may produce symptoms simulating true asthenopia, but these should be carefully distinguished from those resulting from errors of refraction. Epilepsy, chorea, and so forth, have been thought to result from these conditions. With that doctrine, after patient and careful investigation, I have no sympathy. I shall teach that there are no reflex symptoms from the eyes, unless the eyes themselves give warning that they are not doing their work properly. Latent errors of refraction have very little to do, as a rule, in my opinion, even in the causation of asthenopia, and nothing whatever in the production of constitutional disease.

Although I formerly accepted the ordinary classification of asthenopia dependent upon insufficiency of the external ocular muscles, I have come finally to reject it altogether. Of course, I do not deny the existence of insufficiencies of the interni, chiefly in myopia, and of the externi, principally in hypermetropia, nor do I deny that there are many eyes whose external muscles are not capable of doing the average degree of work, but I hold that all these conditions depend on static, fixed conditions of the eyeball ; that they are direct consequences and should not be denied a special nomenclature, but should be classed under the heading of asthenopia occurring in myopia, hypermetropia, hypermetropic astigmatism, and so forth, from faulty conformation of the eye. I formerly measured the relative power of the muscles, but, as has been shown in another part of this book, this relative power varies in different individuals, who have no trouble with their eyes, and I therefore no longer measure it, any more than does the surgeon the relative contractile power of the fingers, legs or arms. Physiologically, it may be interesting, but it can do nothing toward a proper treatment. (Page 450.)

In another place he says:

Muscular asthenopia has played a great part in the diagnosis of refractive cases in our country. One of the believers in great and widespread influence of the eye upon neuroses, abandoned the idea that hypermetropia was the cause of epilepsy and chorea, "more than all other causes combined," to urge that uncorrected insufficiencies of the external muscles was the real cause. This author (G. T. Stevens: Functional Nervous Diseases) has had many followers. Most of them have not gone so far as he, but there has been, and continues to be, an extensive practice of tenotomy and use of prisms to correct defects, which, in my opinion, after a very careful examination continued for years, depend upon errors of refraction, if disturbing, and which may exist, and do exist, in persons who have no asthenopia, and no neuroses, such as chorea and epilepsy. I formerly practised tenotomy, to a limited degree, for latent insufficiencies, and prescribed prisms very frequently. The thorough use of atropia soon, in great part, convinced me of the complete dependence of muscular asthenopia upon errors of refraction, chiefly upon astigmatism, and I gradually ceased almost entirely to prescribe prisms, having found them sometimes beneficial but almost always makeshifts, and poor substitutes for cylindric glasses. When I became familiar with the use of the ophthalmometer, in 1888, I once for all abandoned the idea of relieving muscular asthenopia, except by correction of an error of refraction, and I advised, as I do now, that the term be given up as having no rightful place, as generally used, in ophthalmology. If the student will carefully correct the errors of refraction, he will have no need for tenotomies, except for strabismus, nor for prisms, except for paresis or paralysis of the external muscles. (Page 519.)

The author expresses the utmost confidence in the use of the ophthalmometer in correcting astigmatism, and gives a detailed description of his method of using it. Few ophthalmologists will share with Dr. Roosa the same confidence, but all will admit the great utility of this instrument in suggesting the approximate amount and direction of corneal astigmatism.

In connection with the subject of ocular hygiene, Dr. Roosa utters a most forcible warning and appeal, and it is so pertinent and so just that we quote him somewhat at length:

The demands upon eyes in our days have greatly increased over those of our ancestors. There are many more books, magazines and newspapers to read. Mechanical employment upon fine objects have become more common in this country as our population has grown denser. Besides all this the printers and publishers of the world have everywhere availed themselves of the fact that glasses assist very much in vision and have taken liberties with the public in the way of printing in fine types and in the use of old plates, which give indistinct impressions on the retina and make great demand upon the eyes. This latter state of things is one that every physician should combat. Books should always be well printed, type should always be clear, no matter if by dint of spectacles much can be read that our ancestors, with their poor knowledge of the resources in glass lenses, would never have attempted. Then again, the demands upon school children's eyes have been excessively increased, in the United States at least, in the last fifty years. Children in public schools who will never be able to spend but a few years in study are compelled to take up an inordinate number of varied pursuits and to spend altogether too many hours over books. The practice of writing exercises has been largely multiplied in some of the public schools in New York City and in the cities of this state. These so-called advances in the methods of teaching involve excessive use of the eyes under, sometimes, very unfavorable conditions, that is to say, over desks of inappropriate height for the scholars, in rooms poorly lighted, with air that is sometimes foul for hours. Children are given too many studies to work out at home. It is idle to think that any science can combat such conditions as these. The conditions themselves must be changed or we shall find a race of myopes and asthenopes growing up where formerly we had a strong-eyed people. This is an evil that is beginning to be appreciated in some of the private schools. But some of the teachers in the land seem to be natural enemies, not of the doctor as one of them once suggested, but of the laws of hygiene, which the doctor endeavors to enforce.

In what is said in these chapters on the matter of relieving weak sight with glasses, let it be fully understood that I never, for an instant, condone bad habits which often force glasses upon young people prematurely and which cause evils that they can only imperfectly remedy. There is many a hyperopic or astigmatic person who would never use glasses until presbyopia came on if the eyes were used under favorable conditions. If one were to put down on paper the number of hours that young children attending many schools are compelled to use their eyes on close objects, the table would appear like an exaggeration. I plead earnestly, therefore, that physicians will constantly bear in mind the fact that eyes, especially the eyes of young people, like brains and muscles of the human body may be overtaxed. (Page 439.)

In conclusion, we desire to commend this work to the careful consideration of physicians, first, because its subject-matter is comprehensively and attractively presented; second, because it represents the matured views of an experienced man which are at variance with those of some other ophthalmologists, and may serve as a wholesome check to the propagation of certain extreme tenets and practices that have already gained more or less currency and endorsement in the profession at large. A. A. H.

---

BLOOD SERUM THERAPY AND ANTITOXINS. By GEORGE E. KRIEGER, M. D., Surgeon to the Chicago Hospital, etc. Chicago: E. H. Colegrove & Co. 1895.

The subject of which this book treats is attracting considerable attention at the present time. This is specially the case with reference to the antitoxin treatment of diphtheria. We hear a good deal about Behring's law, which is here given, and furnishes the basis for the application of blood serum to the treatment of disease. It is here asserted that Behring "established the fact that the blood and blood serum of an individual, which has been artificially rendered immune against a certain infectious disease, may be transferred to another individual with the effect to render the latter also immune, no matter how susceptible this animal is to the disease in question."

If this "law" is accepted, everything treated of in the book becomes easily reconcilable. It is a well-written little treatise, and should command the attention of bacteriologists and clinicians.

---

A PRACTICAL MANUAL OF MENTAL MEDICINE. By DR. E. REGIS, Professor of Mental Diseases, Faculty of Medicine, Bordeaux, formerly Chief of Clinique of Mental Diseases, Faculty of Medicine, Paris. With a preface by M. Benjamin Ball, Clinical Professor of Mental Diseases, Faculty of Medicine, Paris. Second edition, thoroughly revised and largely rewritten. Authorized translation by H. M. Bannister, A. M., M. D., late Senior Assistant Physician Illinois Eastern Hospital for the Insane. With introduction by the author. Pp. xvi.—692. Utica, N. Y.: Press of American Journal of Insanity. 1894.

This is the first modern French work on mental diseases which has been translated into English.

After reading the book we are convinced that the American medical profession is truly indebted to Dr. Bannister for his enterprise. The special features of the book are, first, an interesting historical review of insanity, considered in four epochs—namely, first, primitive epoch; second, (*a*) Hippocratic period, (*b*) Alexandrian period, (*c*) Græco-Roman period from Asclepiades to Galen; third epoch, (*a*) the middle ages, (*b*) the renaissance; fourth epoch begins with Pinel. Following the historical sketch is a section on general pathology. The description of various types of insanity—the terminations and complications are all fully and carefully treated.

The chapter on functional elements takes up in detail, first, the disorders of general activity, as excitement and depression; second, disorders of the psychic sphere, as (*a*) of the intellect, (*b*) the emotions, (*c*) of the motor impulses ; third, disorders of the nervous functions—sleep, sensibility, motility.

Disorders of the vegetative functions, circulation, respiration, nutrition and assimilation, the secretions, the temperature, and the trophic and vaso-motor functions.

This chapter is largely physiological in character, and contains much that is not found in other books on mental diseases.

There are also special chapters on heredity, monstrosities, conjugal relations of the insane, which add much to the literature of these subjects.

In the rear of the volume is a good therapeutic formulary. Altogether, we are much pleased with the work and can find little therein to condemn, but, on the contrary, a great deal to praise.

J. W. P.

### BOOKS RECEIVED.

Twentieth Century Practice. An International Encyclopedia of Modern Medical Science. By Leading Authors of Europe and America. Edited by Thomas L. Stedman, M. D., New York City. In twenty volumes. Volume I. Diseases of the Uropoietic System. New York : William Wood & Co. 1895.

A Manual of Diseases of the Ear, for the use of Students and Practitioners of Medicine. By Albert H. Buck, M. D., Clinical Professor of the Diseases of the Ear, College of Physicians and Surgeons ; Columbia College, New York ; Consulting Aural Surgeon, New York Eye and Ear Infirmary and the Presbyterian Hospital. Second revised edition, one volume, post octavo, 467 pages, illustrated, with blank memoranda pages at the back, extra muslin, price, $2.50 New York : William Wood & Co. 1895.

Clinical Gynecology, Medical and Surgical, for Students and Practitioners. By eminent American teachers. Edited by John M. Keating, M. D., LL. D. and by Henry C. Coe, M. D., M. R. C. S., Professor of Gynecology, New York Polyclinic. Illustrated. Octavo, pp. xviii.—994. Philadelphia : J. B. Lippincott Co. 1895.

Materia Medica and Therapeutics for Physicians and Students. By John B. Biddle, M. D., late Professor of Materia Medica and General Therapeutics in the Jefferson Medical College, Philadelphia. Thirteenth edition, revised, re-arranged and enlarged, with special reference to therapeutics, toxicology, the physiological action of medicines and containing all the preparations and remedies described in the United States Pharmacopeia of 1890, to which the work has been made to conform. By Clement Biddle, M. D., Medical Corps, United States Navy. With numerous illustrations. Octavo, pp. 714. Price, $4.00. Philadelphia : P. Blakiston, Son & Co., 1012 Walnut street. 1895.

Relations of Diseases of the Eye to General Diseases. By Max Knies, M. D., Professor Extraordinary at the University of Freidburg. Forming a supplementary volume to every manual and text-book of practical medicine and ophthalmology. Edited by Henry D. Noyes, A. M., M. D., Professor of Ophthalmology and Otology in Bellevue Hospital Medical College ; Executive Surgeon to the New York Eye and Ear Infirmary, etc. Octavo, pp. x.—467. New York : William Wood & Co. 1895.

International Clinics. A Quarterly of Clinical Lectures on Medicine, Neurology, Surgery, Genito-urinary Surgery, Gynecology, Pharyngology, Rhinology, Ophthalmology, Laryngology, Obstetrics, Otology and Dermatology. By professors and lecturers in the leading medical colleges of the United States, Great Britain and Canada. Edited by Judson Daland, M. D., (Univ. of Penna.,) Philadelphia, Instructor in Clinical Medicine and Lecturer on Physical Diagnosis in the University of Pennsylvania ; Assistant Physician to the University Hospital ; Physician to the Philadelphia Hospital and Fellow College of Physicians, Philadelphia. J. Mitchell Bruce, M. D., F. R. C. P., London, England, Physician to and Lecturer on Therapeutics at the Charing Cross Hospital. David W. Finlay, M. D., F. R. C. P., Aberdeen, Scotland, Professor of Practice of Medicine in the University of Aberdeen ; Physician to, and Lecturer on, Clinical Medicine in the Aberdeen Royal Infirmary ; Consulting Physician to the Royal Hospital for Diseases of the Chest, London. Volume IV Fourth series. 1895. Royal 8vo, pp. x.—365. Philadelphia : J. B. Lippincott Co. 1895.

History of Education in Maryland. By Bernard C. Steiner, Ph. D. (J. H. U.), Librarian of the Enoch Pratt Free Library, of Baltimore City, Associate in History Johns Hopkins University. Washington : Government Printing Office. 1894.

Antisepsis and Antiseptics. By Charles Milton Buchanan, M. D., Professor of Chemistry, Toxicology and Metallurgy, National University, Washington, D. C., with an introduction by Professor Augustus C. Bernays. Duodecimo, pp. 368. The Terhune Company : Newark, N. J. 1895.

Dose-Book and Manual of Prescription-Writing, with a list of the official drugs and preparations and also many of the newer remedies now frequently used, with their doses. By E. Q. Thornton, M. D., Demonstrator of Therapeutics, Jefferson Medical College, Philadelphia. Saunders' New Aid Series. Small 8vo, pp. 334. Philadelphia : W. B. Saunders. 1895. Price, cloth, $1.25 net.

